# 1-[(*Z*)-1-Ferrocenyl­ethyl­idene]thio­carbonohydrazide

**DOI:** 10.1107/S1600536812044078

**Published:** 2012-11-03

**Authors:** Abdullah M. Asiri, Muhammad Nadeem Arshad, Muhammad Ishaq, Khalid A. Alamry, Muhammad Shafiq

**Affiliations:** a Chemistry Department & Center of Excellence for Advanced Materials Research (CEAMR), Faculty of Science, King Abdulaziz University, PO Box 80203, Jeddah 21589, Saudi Arabia; b Chemistry Department, Faculty of Science, King Abdulaziz University, PO Box 80203, Jeddah 21589, Saudi Arabia; c Department of Chemistry, Government College University, Faisalabad 38040, Pakistan.

## Abstract

In the title compound, [Fe(C_5_H_5_)(C_8_H_11_N_4_S)], the cyclo­penta­dienyl (Cp) rings of the ferrocene unit are close to being eclipsed. They are inclined to one another at an angle of 1.95 (2)° and lie 3.309 (2)Å away from each other. The ethyl­idene­thio­carbonohydrazide fragment is planar, with an r.m.s. deviation of 0.0347 (2) Å from the mean plane of its eight non-H atoms, and makes dihedral angles of 21.78 (1) and 19.97 (1)° with respect to the two Cp rings. The mol­ecule adopts a *trans* geometry about the C=N double bond. In the crystal, N—H⋯(N/S) and C—H⋯S inter­actions stack the mol­ecules in an inverse fashion along the *b* axis.

## Related literature

For the biological activities of related ferrocene compounds, see: Ornelas (2011 ▶). For related structures, see: Li & Du (2011 ▶); Vikneswaran *et al.* (2010*a* ▶,*b* ▶).
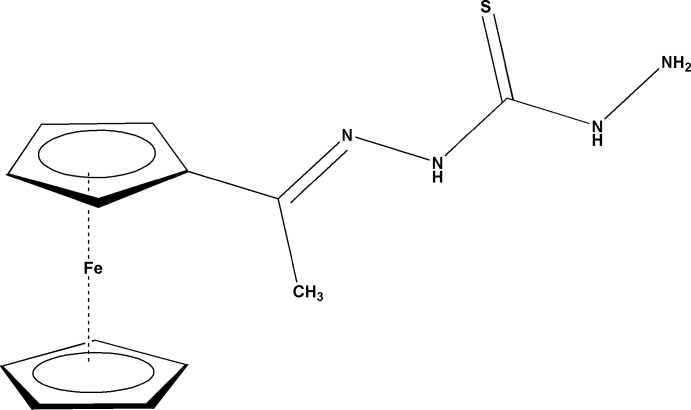


## Experimental

### 

#### Crystal data

[Fe(C_5_H_5_)(C_8_H_11_N_4_S)]
*M* *_r_* = 316.21
Monoclinic, 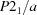

*a* = 6.4560 (2) Å
*b* = 13.0664 (3) Å
*c* = 15.8559 (4) Å
β = 91.028 (2)°
*V* = 1337.34 (6) Å^3^
*Z* = 4
Cu *K*α radiation
μ = 10.47 mm^−1^
*T* = 296 K
0.21 × 0.12 × 0.06 mm

#### Data collection

Agilent SuperNova CCD diffractometer
Absorption correction: multi-scan (*CrysAlis PRO*; Agilent, 2012 ▶) *T*_min_ = 0.435, *T*_max_ = 1.000
8219 measured reflections
2654 independent reflections
2199 reflections with *I* > 2σ(*I*)
*R*_int_ = 0.044

#### Refinement

*R*[*F* ^2^ > 2σ(*F* ^2^)] = 0.053
*wR*(*F* ^2^) = 0.155
*S* = 1.06
2654 reflections
185 parameters
H atoms treated by a mixture of independent and constrained refinement
Δρ_max_ = 0.90 e Å^−3^
Δρ_min_ = −0.53 e Å^−3^

### 

Data collection: *CrysAlis PRO* (Agilent, 2012 ▶); cell refinement: *CrysAlis PRO*; data reduction: *CrysAlis PRO*; program(s) used to solve structure: *SHELXS97* (Sheldrick, 2008 ▶); program(s) used to refine structure: *SHELXL97* (Sheldrick, 2008 ▶); molecular graphics: *PLATON* (Spek, 2009 ▶) and *Mercury* (Macrae *et al.*, 2008 ▶); software used to prepare material for publication: *WinGX* (Farrugia, 1999 ▶) and *X-SEED* (Barbour, 2001 ▶).

## Supplementary Material

Crystal structure: contains datablock(s) I, global. DOI: 10.1107/S1600536812044078/sj5273sup1.cif

Structure factors: contains datablock(s) I. DOI: 10.1107/S1600536812044078/sj5273Isup2.hkl

Additional supplementary materials:  crystallographic information; 3D view; checkCIF report

## Figures and Tables

**Table 1:** Hydrogen-bond geometry (Å, °)

*D*—H⋯*A*	*D*—H	H⋯*A*	*D*⋯*A*	*D*—H⋯*A*
N2—H2*N*⋯N4^i^	0.85 (5)	2.51 (5)	3.349 (5)	170 (4)
N4—H4*A*⋯S1^ii^	0.86 (4)	2.65 (4)	3.501 (3)	169 (4)
N4—H4*B*⋯N1^iii^	0.91 (4)	2.62 (4)	3.443 (4)	151 (3)
C6—H6⋯S1^iv^	0.98	2.81	3.671 (4)	147
N3—H3*N*⋯N1	1.04 (4)	2.09 (4)	2.565 (4)	105 (3)

Symmetry codes: (i) 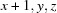; (ii) 
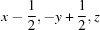
; (iii) 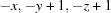; (iv) 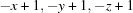.

**Fig. 1.**
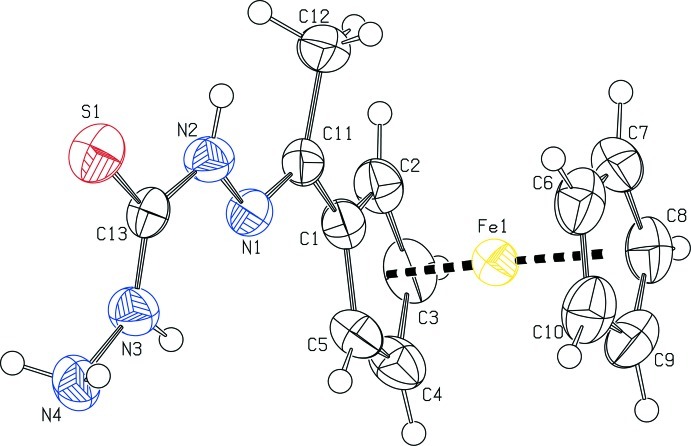
The molecular structure of (I) with 50% displacement ellipsoids.

**Fig. 2.**
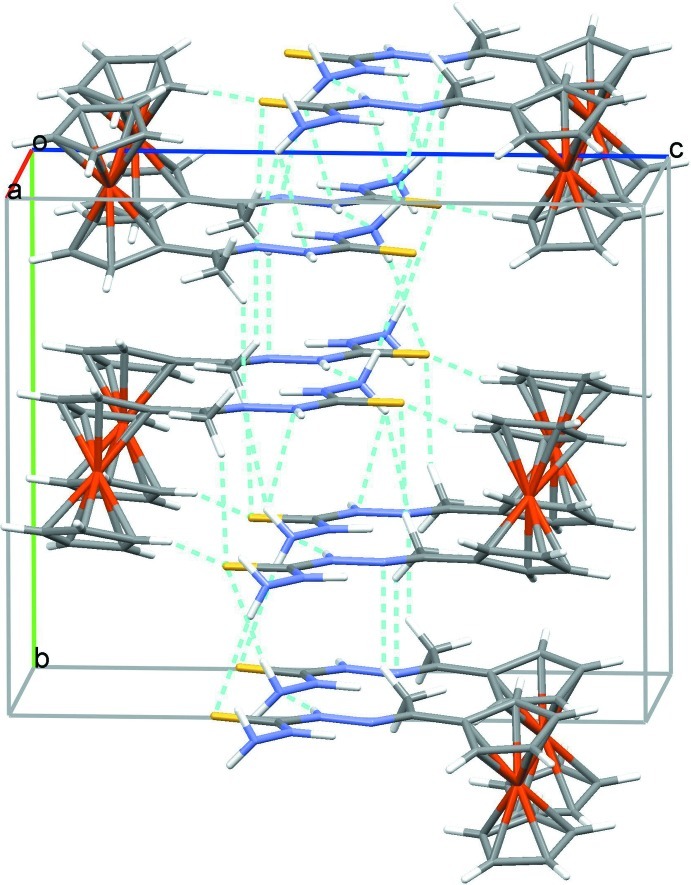
Unit cell packing diagram showing intermolecular hydrogen bonds, drawn using dashed lines.

## supplementary crystallographic information

### Comment

Functionalized ferrocene based organometallic compounds have gained much interest in different areas of applied research especially for their use as anti-tumor agents (Ornelas, 2011). Here we report the crystal structure of the title compound, which is closely related to the reported ferrocene derivatives *N*'-(4-hydroxybenzylidene)ferrocene-1-carbohydrazide (Li & Du, 2011), ferrocene-1-carbaldehyde thiosemicarbazone (Vikneswaran *et al.*, 2010*a*) & ferrocene-1-carbaldehyde 4-ethylthiosemicarbazone (Vikneswaran *et al.*, 2010*b*).

In the title compound, the distances of the Fe1 atom from the centroids of each Cp ring are 1.649 (3) Å Cp1 (C1—C5) & 1.661 (5) Å Cp2 (C5—C10). The dihedral angle between the mean planes of the two Cp rings is 1.95 (2)° and the rings are approximately eclipsed. The mean plane of (ethylidene)thiocarbonohydrazide is twisted 21.78 (1)°, 19.97 (1)° with respect to the Cp1 & Cp2. In the crystal structure intermolecular N—H···(N/S) & C—H···S hydrogen bonding interactions (Table. 1, Fig. 2) stack the molecules in an inverse fashions along the *b* axis.

### Experimental

Thiocarbonohydrazide was refluxed with an equimolar amount of 1-acetylferrocene for 3 h in ethanol to give an orange yellow solution. The volume was reduced under vacum and on cooling gave the title compound as a very stable orange-yellow solid. This was recrystallized from dichloromethane under slow evaporation to obtain suitable orange crystals.

### Refinement

All the C—H H-atoms were positioned with idealized geometry with C—H = 0.93 Å for Cp, & C—H = 0.96 Å for methyl groups. H-atoms were refined as riding with *U*_iso_(H) = *U*_eq_(C), where k = 1.2 for Cp and 1.5 for methyl H-atoms.

All H atoms bound to N were located in a difference Fourier map and refined freely with N—H = 0.85 (5)—1.04 (4) Å, *U*_iso_(H) = 1.2 *U*_eq_(N).

### Crystal data

**Table:**

[Fe(C_5_H_5_)(C_8_H_11_N_4_S)]	*F*(000) = 656
*M**_r_* = 316.21	*D*_x_ = 1.571 Mg m^−^^3^
Monoclinic, *P*2_1_/*a*	Cu *K*α radiation, λ = 1.54184 Å
Hall symbol: -P 2yab	Cell parameters from 3298 reflections
*a* = 6.4560 (2) Å	θ = 2.8–74.4°
*b* = 13.0664 (3) Å	µ = 10.47 mm^−^^1^
*c* = 15.8559 (4) Å	*T* = 296 K
β = 91.028 (2)°	Prismatic, orange
*V* = 1337.34 (6) Å^3^	0.21 × 0.12 × 0.06 mm
*Z* = 4	

### Data collection

**Table:**

Agilent SuperNova (Dual, Cu at zero, Atlas) CCD diffractometer	2654 independent reflections
Radiation source: SuperNova (Cu) X-ray Source	2199 reflections with *I* > 2σ(*I*)
Mirror monochromator	*R*_int_ = 0.044
ω scans	θ_max_ = 74.6°, θ_min_ = 2.8°
Absorption correction: multi-scan (*CrysAlis PRO*; Agilent, 2012)	*h* = −7→8
*T*_min_ = 0.435, *T*_max_ = 1.000	*k* = −15→16
8219 measured reflections	*l* = −16→19

### Refinement

**Table:**

Refinement on *F*^2^	Primary atom site location: structure-invariant direct methods
Least-squares matrix: full	Secondary atom site location: difference Fourier map
*R*[*F*^2^ > 2σ(*F*^2^)] = 0.053	Hydrogen site location: inferred from neighbouring sites
*wR*(*F*^2^) = 0.155	H atoms treated by a mixture of independent and constrained refinement
*S* = 1.06	*w* = 1/[σ^2^(*F*_o_^2^) + (0.1061*P*)^2^ + 0.0547*P*] where *P* = (*F*_o_^2^ + 2*F*_c_^2^)/3
2654 reflections	(Δ/σ)_max_ = 0.001
185 parameters	Δρ_max_ = 0.90 e Å^−^^3^
0 restraints	Δρ_min_ = −0.53 e Å^−^^3^

### Special details

**Table:**

Geometry. All esds (except the esd in the dihedral angle between two l.s. planes) are estimated using the full covariance matrix. The cell esds are taken into account individually in the estimation of esds in distances, angles and torsion angles; correlations between esds in cell parameters are only used when they are defined by crystal symmetry. An approximate (isotropic) treatment of cell esds is used for estimating esds involving l.s. planes.
Refinement. Refinement of F^2^ against ALL reflections. The weighted R-factor wR and goodness of fit S are based on F^2^, conventional R-factors R are based on F, with F set to zero for negative F^2^. The threshold expression of F^2^ > 2sigma(F^2^) is used only for calculating R-factors(gt) etc. and is not relevant to the choice of reflections for refinement. R-factors based on F^2^ are statistically about twice as large as those based on F, and R- factors based on ALL data will be even larger.

### Fractional atomic coordinates and isotropic or equivalent isotropic displacement parameters (Å^2^)

**Table:**

	*x*	*y*	*z*	*U*_iso_*/*U*_eq_	
Fe1	0.25938 (7)	0.50404 (3)	0.15149 (3)	0.0371 (2)	
S1	0.17096 (16)	0.37123 (7)	0.62411 (5)	0.0523 (3)	
N1	0.1806 (4)	0.38168 (19)	0.37809 (17)	0.0384 (6)	
N2	0.2477 (5)	0.3779 (2)	0.46126 (18)	0.0415 (6)	
N3	−0.0929 (5)	0.3759 (2)	0.49333 (19)	0.0456 (7)	
N4	−0.2709 (5)	0.3764 (3)	0.5435 (2)	0.0506 (7)	
C1	0.2337 (5)	0.3837 (2)	0.2332 (2)	0.0399 (7)	
C2	0.3377 (6)	0.3530 (3)	0.1576 (2)	0.0461 (8)	
H2	0.4784	0.3251	0.1548	0.055*	
C3	0.2023 (7)	0.3694 (3)	0.0887 (2)	0.0531 (9)	
H3	0.2333	0.3557	0.0295	0.064*	
C4	0.0139 (6)	0.4114 (3)	0.1191 (2)	0.0524 (9)	
H4	−0.1070	0.4311	0.0845	0.063*	
C5	0.0333 (5)	0.4206 (3)	0.2077 (2)	0.0456 (7)	
H5	−0.0724	0.4476	0.2453	0.055*	
C6	0.4169 (8)	0.6184 (3)	0.2136 (3)	0.0606 (11)	
H6	0.4752	0.6149	0.2710	0.073*	
C7	0.5197 (7)	0.5906 (3)	0.1381 (3)	0.0609 (10)	
H7	0.6617	0.5647	0.1345	0.073*	
C8	0.3836 (7)	0.6080 (3)	0.0701 (3)	0.0567 (9)	
H8	0.4130	0.5963	0.0105	0.068*	
C9	0.1983 (7)	0.6459 (3)	0.1024 (3)	0.0595 (10)	
H9	0.0750	0.6646	0.0688	0.071*	
C10	0.2169 (8)	0.6521 (3)	0.1906 (3)	0.0614 (10)	
H10	0.1104	0.6763	0.2292	0.074*	
C11	0.3164 (5)	0.3790 (2)	0.3196 (2)	0.0389 (7)	
C12	0.5448 (6)	0.3701 (3)	0.3352 (2)	0.0512 (8)	
H12A	0.5964	0.4328	0.3592	0.077*	
H12B	0.6125	0.3570	0.2829	0.077*	
H12C	0.5722	0.3148	0.3737	0.077*	
C13	0.1014 (5)	0.3755 (2)	0.5219 (2)	0.0385 (7)	
H4A	−0.273 (6)	0.318 (3)	0.569 (3)	0.046*	
H3N	−0.116 (6)	0.380 (3)	0.428 (3)	0.046*	
H4B	−0.250 (6)	0.426 (3)	0.583 (3)	0.046*	
H2N	0.375 (7)	0.378 (3)	0.476 (3)	0.046*	

### Atomic displacement parameters (Å^2^)

**Table:**

	*U*^11^	*U*^22^	*U*^33^	*U*^12^	*U*^13^	*U*^23^
Fe1	0.0375 (3)	0.0404 (3)	0.0333 (3)	−0.00125 (18)	0.0015 (2)	−0.00105 (17)
S1	0.0663 (6)	0.0548 (5)	0.0357 (4)	0.0002 (4)	−0.0060 (4)	−0.0019 (3)
N1	0.0401 (14)	0.0415 (13)	0.0337 (12)	0.0000 (10)	−0.0007 (10)	0.0027 (10)
N2	0.0377 (15)	0.0476 (16)	0.0391 (14)	0.0008 (11)	−0.0026 (11)	0.0017 (11)
N3	0.0402 (16)	0.0554 (17)	0.0412 (15)	−0.0007 (12)	0.0024 (11)	0.0054 (12)
N4	0.0487 (18)	0.0489 (17)	0.0547 (19)	−0.0019 (13)	0.0149 (14)	0.0061 (14)
C1	0.0407 (17)	0.0364 (15)	0.0428 (17)	−0.0035 (12)	0.0067 (13)	0.0007 (12)
C2	0.0477 (19)	0.0410 (16)	0.0501 (19)	−0.0010 (14)	0.0126 (15)	−0.0041 (14)
C3	0.065 (2)	0.052 (2)	0.0422 (18)	−0.0127 (17)	0.0075 (16)	−0.0104 (15)
C4	0.0442 (19)	0.068 (2)	0.0451 (19)	−0.0142 (16)	−0.0050 (15)	−0.0037 (16)
C5	0.0351 (16)	0.0561 (19)	0.0456 (18)	−0.0076 (14)	0.0023 (13)	−0.0026 (15)
C6	0.083 (3)	0.048 (2)	0.051 (2)	−0.0168 (18)	−0.017 (2)	0.0041 (15)
C7	0.047 (2)	0.051 (2)	0.085 (3)	−0.0084 (16)	0.0028 (19)	0.007 (2)
C8	0.073 (3)	0.052 (2)	0.0451 (18)	−0.0113 (18)	0.0085 (17)	0.0073 (15)
C9	0.069 (3)	0.051 (2)	0.058 (2)	0.0077 (18)	−0.0071 (19)	0.0122 (17)
C10	0.082 (3)	0.0412 (18)	0.061 (2)	0.0059 (18)	0.011 (2)	−0.0030 (16)
C11	0.0378 (16)	0.0328 (14)	0.0462 (17)	−0.0005 (11)	0.0052 (13)	0.0050 (12)
C12	0.0387 (18)	0.060 (2)	0.055 (2)	0.0031 (15)	0.0037 (15)	0.0093 (16)
C13	0.0471 (18)	0.0295 (13)	0.0389 (15)	0.0005 (12)	0.0007 (13)	0.0011 (11)

### Geometric parameters (Å, º)

**Table:**

Fe1—C2	2.040 (3)	C1—C11	1.463 (5)
Fe1—C5	2.040 (3)	C2—C3	1.403 (6)
Fe1—C7	2.040 (4)	C2—H2	0.9800
Fe1—C1	2.046 (3)	C3—C4	1.426 (6)
Fe1—C9	2.046 (4)	C3—H3	0.9800
Fe1—C8	2.047 (4)	C4—C5	1.414 (5)
Fe1—C6	2.050 (4)	C4—H4	0.9800
Fe1—C3	2.052 (4)	C5—H5	0.9800
Fe1—C10	2.052 (4)	C6—C10	1.405 (7)
Fe1—C4	2.052 (4)	C6—C7	1.427 (6)
S1—C13	1.674 (3)	C6—H6	0.9800
N1—C11	1.288 (4)	C7—C8	1.396 (7)
N1—N2	1.381 (4)	C7—H7	0.9800
N2—C13	1.360 (4)	C8—C9	1.400 (6)
N2—H2N	0.85 (5)	C8—H8	0.9800
N3—C13	1.326 (5)	C9—C10	1.405 (6)
N3—N4	1.409 (4)	C9—H9	0.9800
N3—H3N	1.04 (4)	C10—H10	0.9800
N4—H4A	0.86 (4)	C11—C12	1.495 (5)
N4—H4B	0.91 (4)	C12—H12A	0.9600
C1—C5	1.431 (5)	C12—H12B	0.9600
C1—C2	1.442 (4)	C12—H12C	0.9600
			
C2—Fe1—C5	68.93 (14)	C3—C2—H2	125.9
C2—Fe1—C7	109.73 (16)	C1—C2—H2	125.9
C5—Fe1—C7	159.92 (18)	Fe1—C2—H2	125.9
C2—Fe1—C1	41.34 (12)	C2—C3—C4	108.7 (3)
C5—Fe1—C1	41.02 (14)	C2—C3—Fe1	69.5 (2)
C7—Fe1—C1	124.65 (17)	C4—C3—Fe1	69.7 (2)
C2—Fe1—C9	160.19 (17)	C2—C3—H3	125.7
C5—Fe1—C9	121.08 (17)	C4—C3—H3	125.7
C7—Fe1—C9	67.13 (18)	Fe1—C3—H3	125.7
C1—Fe1—C9	156.89 (16)	C5—C4—C3	107.9 (3)
C2—Fe1—C8	124.93 (15)	C5—C4—Fe1	69.3 (2)
C5—Fe1—C8	157.19 (17)	C3—C4—Fe1	69.6 (2)
C7—Fe1—C8	39.95 (18)	C5—C4—H4	126.1
C1—Fe1—C8	161.13 (17)	C3—C4—H4	126.1
C9—Fe1—C8	40.00 (18)	Fe1—C4—H4	126.1
C2—Fe1—C6	124.23 (17)	C4—C5—C1	108.4 (3)
C5—Fe1—C6	122.18 (16)	C4—C5—Fe1	70.3 (2)
C7—Fe1—C6	40.82 (19)	C1—C5—Fe1	69.71 (18)
C1—Fe1—C6	107.53 (15)	C4—C5—H5	125.8
C9—Fe1—C6	67.21 (17)	C1—C5—H5	125.8
C8—Fe1—C6	67.80 (17)	Fe1—C5—H5	125.8
C2—Fe1—C3	40.11 (17)	C10—C6—C7	107.5 (4)
C5—Fe1—C3	68.27 (15)	C10—C6—Fe1	70.0 (2)
C7—Fe1—C3	124.49 (17)	C7—C6—Fe1	69.2 (2)
C1—Fe1—C3	68.43 (14)	C10—C6—H6	126.2
C9—Fe1—C3	124.11 (19)	C7—C6—H6	126.2
C8—Fe1—C3	109.42 (16)	Fe1—C6—H6	126.2
C6—Fe1—C3	160.33 (19)	C8—C7—C6	108.1 (4)
C2—Fe1—C10	158.85 (17)	C8—C7—Fe1	70.3 (2)
C5—Fe1—C10	105.76 (17)	C6—C7—Fe1	69.9 (2)
C7—Fe1—C10	67.87 (18)	C8—C7—H7	126.0
C1—Fe1—C10	121.34 (15)	C6—C7—H7	126.0
C9—Fe1—C10	40.09 (18)	Fe1—C7—H7	126.0
C8—Fe1—C10	67.71 (16)	C7—C8—C9	107.8 (4)
C6—Fe1—C10	40.07 (19)	C7—C8—Fe1	69.8 (2)
C3—Fe1—C10	158.7 (2)	C9—C8—Fe1	70.0 (2)
C2—Fe1—C4	68.34 (16)	C7—C8—H8	126.1
C5—Fe1—C4	40.43 (14)	C9—C8—H8	126.1
C7—Fe1—C4	159.19 (19)	Fe1—C8—H8	126.1
C1—Fe1—C4	68.56 (15)	C8—C9—C10	109.0 (4)
C9—Fe1—C4	107.24 (18)	C8—C9—Fe1	70.0 (2)
C8—Fe1—C4	122.89 (17)	C10—C9—Fe1	70.2 (2)
C6—Fe1—C4	157.60 (18)	C8—C9—H9	125.5
C3—Fe1—C4	40.67 (16)	C10—C9—H9	125.5
C10—Fe1—C4	121.69 (19)	Fe1—C9—H9	125.5
C11—N1—N2	118.7 (3)	C9—C10—C6	107.6 (4)
C13—N2—N1	117.8 (3)	C9—C10—Fe1	69.7 (2)
C13—N2—H2N	119 (3)	C6—C10—Fe1	69.9 (2)
N1—N2—H2N	123 (3)	C9—C10—H10	126.2
C13—N3—N4	125.7 (3)	C6—C10—H10	126.2
C13—N3—H3N	117 (2)	Fe1—C10—H10	126.2
N4—N3—H3N	117 (2)	N1—C11—C1	115.6 (3)
N3—N4—H4A	107 (3)	N1—C11—C12	124.4 (3)
N3—N4—H4B	106 (3)	C1—C11—C12	120.1 (3)
H4A—N4—H4B	108 (4)	C11—C12—H12A	109.5
C5—C1—C2	106.9 (3)	C11—C12—H12B	109.5
C5—C1—C11	126.3 (3)	H12A—C12—H12B	109.5
C2—C1—C11	126.8 (3)	C11—C12—H12C	109.5
C5—C1—Fe1	69.27 (19)	H12A—C12—H12C	109.5
C2—C1—Fe1	69.11 (19)	H12B—C12—H12C	109.5
C11—C1—Fe1	126.4 (2)	N3—C13—N2	115.0 (3)
C3—C2—C1	108.1 (3)	N3—C13—S1	124.5 (3)
C3—C2—Fe1	70.4 (2)	N2—C13—S1	120.5 (3)
C1—C2—Fe1	69.55 (18)		
			
C11—N1—N2—C13	−176.7 (3)	C4—Fe1—C5—C1	119.4 (3)
C2—Fe1—C1—C5	−118.5 (3)	C2—Fe1—C6—C10	−160.6 (2)
C7—Fe1—C1—C5	160.9 (2)	C5—Fe1—C6—C10	−75.5 (3)
C9—Fe1—C1—C5	46.4 (5)	C7—Fe1—C6—C10	118.7 (4)
C8—Fe1—C1—C5	−168.3 (4)	C1—Fe1—C6—C10	−118.2 (3)
C6—Fe1—C1—C5	119.2 (2)	C9—Fe1—C6—C10	37.8 (3)
C3—Fe1—C1—C5	−81.2 (2)	C8—Fe1—C6—C10	81.3 (3)
C10—Fe1—C1—C5	77.6 (3)	C3—Fe1—C6—C10	167.0 (4)
C4—Fe1—C1—C5	−37.4 (2)	C4—Fe1—C6—C10	−42.0 (5)
C5—Fe1—C1—C2	118.5 (3)	C2—Fe1—C6—C7	80.6 (3)
C7—Fe1—C1—C2	−80.6 (3)	C5—Fe1—C6—C7	165.7 (2)
C9—Fe1—C1—C2	164.9 (4)	C1—Fe1—C6—C7	123.1 (3)
C8—Fe1—C1—C2	−49.8 (5)	C9—Fe1—C6—C7	−80.9 (3)
C6—Fe1—C1—C2	−122.3 (2)	C8—Fe1—C6—C7	−37.4 (3)
C3—Fe1—C1—C2	37.3 (2)	C3—Fe1—C6—C7	48.3 (6)
C10—Fe1—C1—C2	−163.9 (2)	C10—Fe1—C6—C7	−118.7 (4)
C4—Fe1—C1—C2	81.1 (2)	C4—Fe1—C6—C7	−160.7 (4)
C2—Fe1—C1—C11	121.0 (4)	C10—C6—C7—C8	0.4 (4)
C5—Fe1—C1—C11	−120.5 (4)	Fe1—C6—C7—C8	60.2 (3)
C7—Fe1—C1—C11	40.4 (4)	C10—C6—C7—Fe1	−59.8 (3)
C9—Fe1—C1—C11	−74.1 (5)	C2—Fe1—C7—C8	121.3 (3)
C8—Fe1—C1—C11	71.2 (6)	C5—Fe1—C7—C8	−156.3 (4)
C6—Fe1—C1—C11	−1.3 (3)	C1—Fe1—C7—C8	165.1 (2)
C3—Fe1—C1—C11	158.3 (3)	C9—Fe1—C7—C8	−37.7 (3)
C10—Fe1—C1—C11	−42.9 (4)	C6—Fe1—C7—C8	−118.8 (4)
C4—Fe1—C1—C11	−157.9 (3)	C3—Fe1—C7—C8	78.9 (3)
C5—C1—C2—C3	−0.9 (4)	C10—Fe1—C7—C8	−81.3 (3)
C11—C1—C2—C3	179.4 (3)	C4—Fe1—C7—C8	40.5 (6)
Fe1—C1—C2—C3	−60.1 (2)	C2—Fe1—C7—C6	−119.9 (3)
C5—C1—C2—Fe1	59.2 (2)	C5—Fe1—C7—C6	−37.5 (6)
C11—C1—C2—Fe1	−120.5 (3)	C1—Fe1—C7—C6	−76.1 (3)
C5—Fe1—C2—C3	80.9 (2)	C9—Fe1—C7—C6	81.1 (3)
C7—Fe1—C2—C3	−120.5 (3)	C8—Fe1—C7—C6	118.8 (4)
C1—Fe1—C2—C3	119.0 (3)	C3—Fe1—C7—C6	−162.3 (2)
C9—Fe1—C2—C3	−43.4 (5)	C10—Fe1—C7—C6	37.5 (3)
C8—Fe1—C2—C3	−78.5 (3)	C4—Fe1—C7—C6	159.3 (4)
C6—Fe1—C2—C3	−163.8 (2)	C6—C7—C8—C9	−0.1 (5)
C10—Fe1—C2—C3	160.0 (4)	Fe1—C7—C8—C9	59.9 (3)
C4—Fe1—C2—C3	37.3 (2)	C6—C7—C8—Fe1	−60.0 (3)
C5—Fe1—C2—C1	−38.2 (2)	C2—Fe1—C8—C7	−78.9 (3)
C7—Fe1—C2—C1	120.4 (2)	C5—Fe1—C8—C7	159.1 (4)
C9—Fe1—C2—C1	−162.4 (5)	C1—Fe1—C8—C7	−40.9 (6)
C8—Fe1—C2—C1	162.5 (2)	C9—Fe1—C8—C7	118.7 (4)
C6—Fe1—C2—C1	77.2 (3)	C6—Fe1—C8—C7	38.2 (3)
C3—Fe1—C2—C1	−119.0 (3)	C3—Fe1—C8—C7	−120.9 (3)
C10—Fe1—C2—C1	40.9 (5)	C10—Fe1—C8—C7	81.7 (3)
C4—Fe1—C2—C1	−81.7 (2)	C4—Fe1—C8—C7	−164.1 (3)
C1—C2—C3—C4	0.7 (4)	C2—Fe1—C8—C9	162.3 (3)
Fe1—C2—C3—C4	−58.8 (3)	C5—Fe1—C8—C9	40.4 (5)
C1—C2—C3—Fe1	59.5 (2)	C7—Fe1—C8—C9	−118.7 (4)
C5—Fe1—C3—C2	−82.7 (2)	C1—Fe1—C8—C9	−159.7 (4)
C7—Fe1—C3—C2	79.7 (3)	C6—Fe1—C8—C9	−80.5 (3)
C1—Fe1—C3—C2	−38.4 (2)	C3—Fe1—C8—C9	120.3 (3)
C9—Fe1—C3—C2	163.7 (2)	C10—Fe1—C8—C9	−37.1 (3)
C8—Fe1—C3—C2	121.6 (2)	C4—Fe1—C8—C9	77.2 (3)
C6—Fe1—C3—C2	43.3 (5)	C7—C8—C9—C10	−0.2 (5)
C10—Fe1—C3—C2	−160.1 (4)	Fe1—C8—C9—C10	59.5 (3)
C4—Fe1—C3—C2	−120.2 (3)	C7—C8—C9—Fe1	−59.8 (3)
C2—Fe1—C3—C4	120.2 (3)	C2—Fe1—C9—C8	−47.2 (6)
C5—Fe1—C3—C4	37.5 (2)	C5—Fe1—C9—C8	−163.0 (2)
C7—Fe1—C3—C4	−160.2 (2)	C7—Fe1—C9—C8	37.7 (3)
C1—Fe1—C3—C4	81.8 (2)	C1—Fe1—C9—C8	163.4 (4)
C9—Fe1—C3—C4	−76.2 (3)	C6—Fe1—C9—C8	82.1 (3)
C8—Fe1—C3—C4	−118.2 (2)	C3—Fe1—C9—C8	−79.5 (3)
C6—Fe1—C3—C4	163.5 (4)	C10—Fe1—C9—C8	120.0 (4)
C10—Fe1—C3—C4	−39.9 (5)	C4—Fe1—C9—C8	−121.0 (3)
C2—C3—C4—C5	−0.3 (4)	C2—Fe1—C9—C10	−167.2 (4)
Fe1—C3—C4—C5	−59.0 (3)	C5—Fe1—C9—C10	77.1 (3)
C2—C3—C4—Fe1	58.7 (3)	C7—Fe1—C9—C10	−82.3 (3)
C2—Fe1—C4—C5	82.5 (2)	C1—Fe1—C9—C10	43.4 (5)
C7—Fe1—C4—C5	171.2 (4)	C8—Fe1—C9—C10	−120.0 (4)
C1—Fe1—C4—C5	37.9 (2)	C6—Fe1—C9—C10	−37.8 (3)
C9—Fe1—C4—C5	−118.0 (2)	C3—Fe1—C9—C10	160.5 (3)
C8—Fe1—C4—C5	−159.0 (2)	C4—Fe1—C9—C10	119.0 (3)
C6—Fe1—C4—C5	−46.2 (5)	C8—C9—C10—C6	0.5 (5)
C3—Fe1—C4—C5	119.3 (3)	Fe1—C9—C10—C6	59.9 (3)
C10—Fe1—C4—C5	−76.6 (3)	C8—C9—C10—Fe1	−59.4 (3)
C2—Fe1—C4—C3	−36.8 (2)	C7—C6—C10—C9	−0.5 (4)
C5—Fe1—C4—C3	−119.3 (3)	Fe1—C6—C10—C9	−59.8 (3)
C7—Fe1—C4—C3	51.9 (5)	C7—C6—C10—Fe1	59.3 (3)
C1—Fe1—C4—C3	−81.4 (2)	C2—Fe1—C10—C9	168.0 (4)
C9—Fe1—C4—C3	122.7 (3)	C5—Fe1—C10—C9	−119.8 (3)
C8—Fe1—C4—C3	81.7 (3)	C7—Fe1—C10—C9	80.3 (3)
C6—Fe1—C4—C3	−165.5 (4)	C1—Fe1—C10—C9	−161.6 (3)
C10—Fe1—C4—C3	164.1 (2)	C8—Fe1—C10—C9	37.0 (3)
C3—C4—C5—C1	−0.3 (4)	C6—Fe1—C10—C9	118.5 (4)
Fe1—C4—C5—C1	−59.5 (2)	C3—Fe1—C10—C9	−49.4 (6)
C3—C4—C5—Fe1	59.2 (3)	C4—Fe1—C10—C9	−78.9 (3)
C2—C1—C5—C4	0.7 (4)	C2—Fe1—C10—C6	49.4 (5)
C11—C1—C5—C4	−179.6 (3)	C5—Fe1—C10—C6	121.6 (3)
Fe1—C1—C5—C4	59.8 (2)	C7—Fe1—C10—C6	−38.2 (3)
C2—C1—C5—Fe1	−59.1 (2)	C1—Fe1—C10—C6	79.9 (3)
C11—C1—C5—Fe1	120.6 (3)	C9—Fe1—C10—C6	−118.5 (4)
C2—Fe1—C5—C4	−80.9 (2)	C8—Fe1—C10—C6	−81.5 (3)
C7—Fe1—C5—C4	−170.9 (4)	C3—Fe1—C10—C6	−168.0 (4)
C1—Fe1—C5—C4	−119.4 (3)	C4—Fe1—C10—C6	162.6 (2)
C9—Fe1—C5—C4	80.0 (3)	N2—N1—C11—C1	−179.7 (3)
C8—Fe1—C5—C4	50.9 (5)	N2—N1—C11—C12	1.1 (4)
C6—Fe1—C5—C4	161.1 (2)	C5—C1—C11—N1	19.8 (5)
C3—Fe1—C5—C4	−37.7 (2)	C2—C1—C11—N1	−160.5 (3)
C10—Fe1—C5—C4	120.7 (3)	Fe1—C1—C11—N1	109.6 (3)
C2—Fe1—C5—C1	38.5 (2)	C5—C1—C11—C12	−161.0 (3)
C7—Fe1—C5—C1	−51.5 (5)	C2—C1—C11—C12	18.6 (5)
C9—Fe1—C5—C1	−160.6 (2)	Fe1—C1—C11—C12	−71.3 (4)
C8—Fe1—C5—C1	170.3 (4)	N4—N3—C13—N2	−178.3 (3)
C6—Fe1—C5—C1	−79.6 (3)	N4—N3—C13—S1	2.3 (4)
C3—Fe1—C5—C1	81.7 (2)	N1—N2—C13—N3	0.8 (4)
C10—Fe1—C5—C1	−119.9 (2)	N1—N2—C13—S1	−179.8 (2)

### Hydrogen-bond geometry (Å, º)

**Table:**

*D*—H···*A*	*D*—H	H···*A*	*D*···*A*	*D*—H···*A*
N2—H2*N*···N4^i^	0.85 (5)	2.51 (5)	3.349 (5)	170 (4)
N4—H4*A*···S1^ii^	0.86 (4)	2.65 (4)	3.501 (3)	169 (4)
N4—H4*B*···N1^iii^	0.91 (4)	2.62 (4)	3.443 (4)	151 (3)
C6—H6···S1^iv^	0.98	2.81	3.671 (4)	147
N3—H3*N*···N1	1.04 (4)	2.09 (4)	2.565 (4)	105 (3)

Symmetry codes: (i) *x*+1, *y*, *z*; (ii) *x*−1/2, −*y*+1/2, *z*; (iii) −*x*, −*y*+1, −*z*+1; (iv) −*x*+1, −*y*+1, −*z*+1.
